# Knowledge, Attitudes, and Practices Related to Uterotonic Drugs during Childbirth in Karnataka, India: A Qualitative Research Study

**DOI:** 10.1371/journal.pone.0062801

**Published:** 2013-04-29

**Authors:** Nitya Nand Deepak, Ellie Mirzabagi, Alissa Koski, Vandana Tripathi

**Affiliations:** 1 PATH, New Delhi, India; 2 Johns Hopkins Bloomberg School of Public Health, Washington, D. C., United States of America; 3 Department of Epidemiology, Biostatistics, and Occupational Health, McGill University, Montreal, Quebec, Canada; 4 Department of Population, Family, and Reproductive Health, Johns Hopkins Bloomberg School of Public Health, Baltimore, Maryland, United States of America; Baylor College of Medicine, United States of America

## Abstract

**Background and Objectives:**

India has the highest annual number of maternal deaths of any country. As obstetric hemorrhage is the leading cause of maternal death in India, numerous efforts are under way to promote access to skilled attendance at birth and emergency obstetric care. Current initiatives also seek to increase access to active management of the third stage of labor for postpartum hemorrhage prevention, particularly through administration of an uterotonic after delivery. However, prior research suggests widespread inappropriate use of uterotonics at facilities and in communities–for example, without adequate monitoring or referral support for complications. This qualitative study aimed to document health providers’ and community members’ current knowledge, attitudes, and practices regarding uterotonic use during labor and delivery in India’s Karnataka state.

**Methods:**

140 in-depth interviews were conducted from June to August 2011 in Bagalkot and Hassan districts with physicians, nurses, recently delivered women, mothers-in-law, traditional birth attendants (*dais*), unlicensed village doctors, and chemists (pharmacists).

**Results:**

Many respondents reported use of uterotonics, particularly oxytocin, for labor augmentation in both facility-based and home-based deliveries. The study also identified contextual factors that promote inappropriate uterotonic use, including high value placed on pain during labor; perceived pressure to provide or receive uterotonics early in labor and delivery, perhaps leading to administration of uterotonics despite awareness of risks; and lack of consistent and correct knowledge regarding safe storage, dosing, and administration of oxytocin.

**Conclusions:**

These findings have significant implications for public health programs in a context of widespread and potentially increasing availability of uterotonics. Among other responses, efforts are needed to improve communication between community members and providers regarding uterotonic use during labor and delivery and to target training and other interventions to address identified gaps in knowledge and ensure that providers and pharmacists have up-to-date information regarding proper usage of uterotonic drugs.

## Introduction

### Global Maternal Mortality and Uterotonic Usage

Hemorrhage, defined as blood loss of 500 ml or more, is a leading cause of maternal death globally [1], [2]. Postpartum hemorrhage (PPH) contributes to a higher proportion of maternal mortality in developing countries, particularly in rural settings with limitations in infrastructure, availability of skilled birth attendants (SBAs), and uterotonics for management of PPH [3], [4]. United Nations Millennium Development Goal 5–to reduce maternal mortality by 75% by 2015 [5]–cannot be reached without addressing PPH [2], [6].

PPH is one of the few obstetric complications for which an effective preventive intervention is available. The active management of the third stage of labor (AMTSL) is a package of interventions including administration of a uterotonic drug immediately following delivery, controlled cord traction, and fundal massage following delivery of the placenta [7]. There may be changes to AMTSL policy and guidance in the near future, given recent research regarding controlled cord traction and the dynamic state of evidence regarding the full package of interventions [8]. However, the World Health Organization (WHO) currently recommends AMTSL for PPH prevention in the presence of an SBA [7].

As half of all births in low-income countries take place at home or in peripheral health facilities [9], there is a need to adapt AMTSL for use outside of hospital settings. In the absence of AMTSL, WHO recommends that a uterotonic drug be offered by a health worker trained in its use for PPH prevention [7].

Oxytocin is the drug of choice for PPH prevention [7], but feasibility of use is limited in many settings because oxytocin is only available in injectable form and requires refrigeration. Recent calls to expand access to oxytocin for PPH prevention have been accompanied by concerns that the drug would also be used inappropriately for induction and augmentation of labor [10], [11], [12]. Labor induction and augmentation should only be performed by highly trained health workers in facilities with access to emergency obstetric care due to increased risks of complications accompanying these procedures. World Health Organization guidance deems inappropriate use of oxytocin (e.g., administration of oxytocin prior to delivery in peripheral health facilities or by low-level health workers) dangerous because the dosage may be difficult to monitor and low-level workers and peripheral facilities may not be able to manage adverse effects [7], [13]. Inappropriate administration may result in hyperstimulation of the uterus, which can lead to uterine rupture, fetal asphyxia, and/or fetal demise [12], [14].

Despite these risks, unmonitored and inappropriate use of uterotonics appears to be common in developing countries, including in up to 69% of home births [15]. Recent studies have documented inappropriate use of uterotonics in South Asia, particularly in India and Bangladesh [10], [16], [17]. Intramuscular (IM) oxytocin injection during the first and second stages of labor can be dangerous because dosing cannot be adjusted in response to the strength of uterine contractions, increasing risks of uterine rupture and harm to the fetus [13]. However, IM oxytocin is reportedly common to induce and augment labor in some settings, including at home births and in health facilities; it has been claimed that IM administration is practiced for rapid delivery at the request of pregnant women or their families [11], [18], [19].

### Maternal Health Policy in India and Karnataka

The maternal mortality ratio is estimated to be between 230 and 254 deaths per 100,000 live births [20], [21]. While this represents a significant decline from previous decades, India has the largest number of maternal deaths in the world, between 50,000 and 63,000 annually [20], [22]. Obstetric hemorrhage contributes to about 37% of maternal deaths in India [23], [24]. Under the National Rural Health Mission, the Government of India has taken several steps to improve maternal health. Three key efforts include encouraging delivery in institutions through monetary incentives from the Janani Suraksha Yojana program, supporting emergency obstetric care development, and training auxiliary nurse-midwives (ANMs) and nurses to gain competencies as SBAs [22], [25], [26]. The National Rural Health Mission approach also includes the Reproductive and Child Health II program, which promises investments in emergency obstetric care provision, including training doctors in emergency skills, upgrading ANM skills, ensuring blood storage points in every district, and upgrading community health centers to meet national standards. It also supports demand-side financing to spur utilization of services through incentives for assisted home deliveries, institutional deliveries, and caesarean sections [22], [27]. To bolster care quality as well as SBA utilization of SBA and institutional delivery, in 2005 the government developed evidence-based guidelines for SBAs–including doctors, ANMs, staff nurses, and lady health volunteers–on care during the antenatal, labor and delivery, and postpartum periods [28]. Current training guidance for SBAs promotes AMTSL, including uterotonic use for PPH prevention and treatment [29].

Karnataka state has implemented a number of maternal and neonatal health programs, including Madilu, which provides women who receive SBA or institutional delivery with a kit of materials to use in the postnatal period; Prasuthi Araike, which provides cash incentives for antenatal and postnatal care; Thayi Bhagya, which provides cash incentives to use obstetric services in private facilities; and the Janani Suraksha Yojana program [30].

Despite increased emphasis on evidence-based medicine in India as a whole and in Karnataka, the inappropriate uterotonic practices described above may remain common. Previous studies of oxytocin use practices in South Asia have called for additional research that contributes to “tracing how pharmaceuticals such as oxytocin are being used “on the ground” and understanding how they are embedded in wider social and economic contexts. However, little information is available regarding current uterotonic use practices in peripheral health facilities. Additionally, the knowledge and attitudes underlying uterotonic use practices are not well understood, particularly given the diversity of cultural and health systems contexts across India. It is important to understand the motivation and context for current uterotonic use if programs to scale up prophylactic use of oxytocin for prevention of PPH are to succeed without exacerbating inappropriate practices.

This exploratory study aimed to address these evidence gaps, particularly to document health care providers’ and community members’ knowledge, attitudes, and practices regarding uterotonic use during labor and delivery from the community to the facility level in Karnataka.

## Materials and Methods

### Ethics Statement

The study protocol was approved by the institutional review boards of the Johns Hopkins Bloomberg School of Public Health and PATH in the United States and of the Health Ministry Screening Committee and the CLINICOM Ethics Committee in Bangalore, India. The project director from the Karnataka Reproductive and Child Health II Project and district health officers from Hassan and Bagalkot provided administrative approval. All study participants provided written informed consent.

### Study Setting

The study took place in Karnataka state, where 65% of deliveries take place in institutions, compared to a national average of 47% [30]. Nearly three-fourths (72%) of women in Karnataka are assisted at delivery by health professionals (including both facility and assisted home births), compared with 53% nationally [31]. However, the rural population is still relatively underserved; only 60% of rural births in Karnataka take place in institutions as compared with 80% in urban areas [31].

The study setting was two districts in Karnataka–Bagalkot in the north and Hassan in the south. These districts were chosen in consultation with state maternal health authorities and vary by indicators such as health infrastructure and institutional deliveries (Table 1); e.g., Hassan has a higher urban institutional delivery rate compared to Bagalkot (80% vs. 47%) [31]. The study districts provide a context of remote services in a rural resource-poor area, typical of many developing-country settings, but with the potential and infrastructure for further improvements in the near future.

**Table 1:** Bagalkot and Hassan District Profiles.

Indicator	Bagalkot District	Hassan District
Population (in thousands)	1,652	1,722
Rural population (% of total)	71%	82.3%
Mothers who had ≥3 antenatal care visits during last pregnancy	63%	94%
Institutional births (% of total)	46.6%	80.3%
Delivery at home with a skilled provider (% of total)	29%	18.8%
Number of Community Health Centers	25	30
Number of Primary Health Centers	10	15
Number of Sub-Centers	31	38

Source: International Institute for Population Sciences 2010 [30].

### Study Methods

This qualitative research study applied a case study approach in two districts with contrasting features. Semi-structured in-depth interviews (IDIs) were conducted from June to August 2011 with respondents identified using purposive and snowball sampling approaches. Ten IDIs were conducted within each of seven respondent cadres, for a total of 140 IDIs:

- Physicians
- Nurses–primarily ANMs, but also staff nurses and lady health volunteers
- Traditional birth attendants, known locally as *dais*
- Unlicensed village doctors, informal providers with no or minimal formal medical training
- Chemists/pharmacists
- Women who delivered within six months prior
- Mothers-in-law with at least one grandchild

Experienced researchers were recruited and further trained in research ethics and qualitative methodology. IDI guides were used to ensure consistency across multiple interviewers; topics are summarized in Table 2.

**Table 2:** Interview Topics by Respondent Cadre.

Respondent Type	Sample Interview Topics/Questions
**Facility Level**	
Physicians (n = 20)	Clinical experience and practice with uterotonics, frequency of use, attitude toward uterotonic use during labor and delivery, availability of uterotonics within the facility
Auxiliary nurse midwives/staff nurses/ladyhealth visitors (n = 20)	Clinical experience with uterotonics, perceived effect of uterotonic substances on labor/delivery processes, attitude toward uterotonic use during labor and delivery
**Community Level**	
Mothers who gave birth within the past six months (n = 20)	Personal experience with use of uterotonics, attitude toward uterotonic use during labor and delivery (e.g., did they request uterotonics? If so, why?), cost associated with purchase/administration of uterotonics
Mothers-in-law with ≥ 1 grandchild (n = 20)	Role in delivery process, attitude toward uterotonic use during labor and delivery, cost associated with purchase and/or administration of uterotonics, personal recollection of uterotonic use practices (e.g., when did it become commonplace?)
Traditional birth attendants, also knownas *dai*s (n = 20)	Perceived effect of uterotonic substances on labor/delivery processes, how effective they believe various substances to be, indications for use, uterotonic stock procurement
Unlicensed health workers, also knownas village doctors (n = 20)	Role in delivery processes at home and in facilities, clinical experience with uterotonics, perceived effect of uterotonic substances on labor/delivery processes, economics of uterotonic administration, stock procurement
Pharmacists, also known as chemists (n = 20)	Stock procurement, pricing, knowledge of prescription policy, selling practices (i.e., any advice, qualifying questions, contraindications)

All IDI guides and consent forms were translated from English into the local language, Kannada. Draft interview guides were pretested and changed based on findings. All IDIs were tape-recorded and transcribed by local researchers using a standardized transcription protocol.

Thematic coding and content analysis was performed using NVivo 8.0 software. Following preparatory transcript review, the authors collaboratively developed an initial codebook that defined themes and was used to code transcripts. The codebook was updated by the research team using an iterative process, as additional transcripts suggested new themes. Codes and coded output were compared within and across participant cadres to identify primary themes and notable agreement or contrasts.

## Results

While the study explored practices and perspectives on numerous aspects of uterotonic use throughout labor and delivery, findings reflect primary foci within these broader topics. The results reported below indicate respondent priorities and the frequency of reported themes. First, findings regarding how uterotonics are perceived and used by formal providers at facilities and by informal providers at the community level are reported. These are followed by descriptions of how uterotonic use is perceived by community members. Similar knowledge, beliefs, perceptions, and uterotonic use practices were reported in both districts where interviews were conducted. Therefore, the findings are not disaggregated by district.

### Use of Uterotonics by Formal Providers at Facilities

#### Physicians

Physicians described a variety of indications for which they used uterotonics. Some stated that they did not use oxytocin to induce or augment labor, while others reported that they do. On being asked what the use of oxytocin was, one physician from Bagalkot reported, *“It is used to induce contractions, while Methergine* [trade name for methylergometrine maleate] *is used to control bleeding.”* Some physicians shared that they used oxytocin to augment labor in the past, but now use it to prevent PPH as part of AMTSL. It appears that other pharmaceuticals are also being used to induce and augment labor earlier in the intrapartum period. Apart from oxytocin, physicians also reported using Methergine and misoprostol tablets for prevention of PPH.

*“… as soon as the pain starts, oxytocin we were using in those days, and even in the second stage we were using it, but now in the first stage we are using Epidosin [trade name for valethamate bromide] and in the third stage we are using oxytocin.” – Bagalkot, Physician*

#### Nurses

ANMs described using several different drugs to augment labor at public health facilities, including oxytocin, Epidosin, and misoprostol. Most ANMs said that oxytocin is used to speed the delivery process, generally following a determination of cervical dilation. Overall, ANMs reported that the decision to administer oxytocin is made by the physician and, in the physician’s absence, by the ANM. To prevent PPH, ANMs reported using oxytocin, Methergine, or misoprostol.

*“We do the pelvic examination first and we see the dilatation and see how much it is and if we feel that it will be normal delivery only then 3 to 4 fingers dilatation is there then we give Pitocin [trade name for synthetic oxytocin] in drip.” - Bagalkot, ANM*

*Whatever we do, we take doctors suggestion and only then we do; in case if the doctors have gone on village visit or if not available only then we take our own decision” – Bagalkot, ANM*

*“First we see the dilatation and if its 1–2 finger dilatation then we wait for 2–3 hours and if dilatation happens and pain is less then we give Epidosin injection and after 5 finger dilatation we give oxytocin through drip.” Bagalkot, ANM*

### Use of Uterotonics by Informal Providers in the Community

Oxytocin is commonly used by informal providers (i.e., village doctors) to augment labor in community settings, particularly if uterine contractions are deemed insufficient.

*“See, oxytocin is given when the uterine contractions are low, and if she doesn’t deliver normally we give Syntocinon [trade name for synthetic oxytocin]; otherwise we don’t give. Later, they may call us if there is long duration of pain … and we just give the Syntocinon…. It increases in contraction and she will deliver with single injection of 5 international units of Syntocinon.” – Hassan, Village Doctor*

*“First we see if the days are complete. Then PV [pelvic] examination, and if there is dilation we give oxytocin in IV drip.” – Bagalkot, Village Doctor*

*“If the opening where the child has to come is too strong, then it has to tear…by this injection it helps in giving too much of pain and it tears…so the child comes out.” – Hassan, Village Doctor*

Many village doctors reported observing similar uterotonic use practices while they were “training” informally under medical doctors and stated that they imitate observed procedures in their own work. Village doctors seem to play a very specific role in deliveries that occur outside of health facilities. Rather than attending a woman throughout labor, they are called by *dais* to administer uterotonic injections. Decision-making in this relationship may vary. Several village doctors suggested that they retained control over the decision to administer oxytocin. However, one village doctor stated that the *dai* is the decision-maker.

*“Now we don’t do [deliveries] here in the clinic; we go to their house and then do the delivery. There are sulagitti [dais] and they do the delivery. They will go and see and then only when they tell the entire details then we will go and see them and only when there is pain they will tell us to give the injection…. They [dais] are very well-versed and… they are more experienced than us.” –* Bagalkot, Village Doctor

### Uterotonic Administration and Dosage Practices

#### Formal providers

Physicians and ANMs reported monitoring cervical dilation, frequency of contractions, and strength of labor pain before and during oxytocin administration. They reported administering further doses based on whether the first dose of oxytocin had no effect, depending on the delivering woman’s condition. Physicians and ANMs reported using the finger method to assess cervical dilation and then, based on that, deciding whether to administer uterotonics.

Oxytocin is generally available in 5-IU ampoules, and facility-based providers commonly administered at least two doses (10 IU) of oxytocin via saline. Formal providers expressed a variety of beliefs and practices regarding when it is appropriate to give oxytocin for augmentation in relation to cervical dilation, and also regarding dosing:

*“We wait for 1-finger or 2-finger dilation. If it’s there then we suggest the patient walk for some time and ask her to wait since dilation is not there, and if it’s 3- or 4-finger dilation, then I give 2.5 units of oxytocin [a unit is 5 IU] for uterine contraction and vaginal dilation….”*

*– Hassan, Physician*

*“…then we add 5 ml of oxytocin, that is around an ampoule of oxytocin; so then we give 10 drops of oxytocin infusion and… she has to go for three contractions in 15 minutes, good contractions in that interval….” – Hassan, Physician*

*“…first we see what sort of dilation is there, then we proceed with further treatment. Means if the dilation is less, then it may take 4–5 hours for delivery to happen, and then after 2 hours we give them an injection and make the delivery happen.” – Bagalkot, ANM*

*“Doctor looks at the condition of the patient and then decides how much to give; it depends on the condition of the patient and we can give from 2 ampoules to 4 ampoules [10 IU to 20 IU]. We see the condition of the patient and then give the quantity required, like if there are no pains then we give 5 units [25 IU]….” – Bagalkot, ANM*

#### Informal providers

Village doctors reported similar oxytocin administration practices, although their reported doses ranged from 10 to 30 IU. Village doctors often relied on *dais’* reports to assess when to administer uterotonics, as noted above. Village doctors also reported being conscious of the need to refer to other providers for additional or surgical care, should the oxytocin not succeed in facilitating delivery. Related to this, one *dai* also reported that village doctors require that *dais* monitor the laboring woman once injectable uterotonics have been used to augment labor:

*“We will first give one ampoule, and if the delivery doesn’t happen with that, then we will shift to another ampoule of dose. If the child doesn’t come out with the second dose also, then we will advise a caesarean and we can’t wait for long, and so we will send them to the lady doctor. We will give the third and the last dose only when we are sure that the child will come with this last dose.” – Bagalkot, Village Doctor*

*I will tell the village doctor to give injection for the pain to come fast. They give two initially and if the pain doesn’t come then they will give four.” – Bagalkot, Dai*

### Uterotonic Storage

Physicians and most ANMs did not know how or under what conditions oxytocin was stored in their facilities. Several ANMs reported that oxytocin did not require refrigeration and could be stored at room temperature. On being questioned about specific drugs like Prostodin and Methergine, one ANM reported that these are stored in refrigerators; the same respondent shared that oxytocin and Epidosin can be stored “outside.”

Similarly, chemists also gave mixed responses; while some reported refrigerating oxytocin, others kept it on the counter.

*“In the refrigerator we stock it; and others, they switch off the refrigerator at night, but we don’t switch it off and it’s on throughout the day.” – Bagalkot, Chemist*

*“Methergine we store in the fridge; oxytocin we store outside.” – Bagalkot, Chemist*

*“We store oxytocin in fridge and Epidosin outside.” – Bagalkot, Chemist*

These statements from chemists illustrate the fact that the majority in this study sample lack any formal training in handling medicines. Interviewed chemists’ work experience in the pharmacy sector ranged from 6 months to 30 years, but only three of ten respondents had formal degree-level training in dispensing and dealing with medicines. The others tended to have trained under family members and/or received short (e.g., 3-month) trainings.

### Formal and Informal Providers’ Perceptions of Uterotonic Risks

While numerous providers described using uterotonics for labor induction and augmentation, a number of formal providers were aware of potential adverse effects of such use, including uterine rupture and fetal asphyxia:

*“We are not supposed to give more than 20 units because the baby may have lot of trouble like stillborn or… mother’s uterus may get ruptured if the quantity is more as far as I know and the mother’s condition will become serious; we have to give only specified units.” – Hassan, ANM*

*“If oxytocin is given beforehand it creates a lot of problems for the baby so it is given when full dilation happens and that too after taking doctor’s permission. If dilation has not happened and if this medicine is given, then the baby can’t come out easily, and due to that, pain increases and it also creates a lot of problems for the baby also.” – Bagalkot, ANM*

*“…utilization of these drugs has not much advantages and they have more of disadvantages because it augments deliveries so either the baby ends up with asphyxia or the mother ends up with PPH….” – Hassan, Physician*

Despite concerns about the above-noted risks, most physicians reported that oxytocin is a safe drug and that they use it during the third stage of labor to prevent PPH. However, physicians also expressed belief that there is rampant misuse of uterotonics by unqualified providers in community settings.

*“Even the quacks are using oxytocin; they don’t know the dosage and they are all giving three to four oxytocin [ampoules] in the drip and her uterus may rupture” – Bagalkot, Physician*

*“If you use uterotonics frequently it has side effects on the uterus and there are some quacks who, for the sake of getting the baby out fast, use uterotonics on an hourly basis, especially oxytocin; and because of that chances are there that the baby might die due to asphyxia and chance of uterus rupture is also more.” – Hassan, Physician*

A few village doctors echoed this perception of risk.

*“…it is only one dose we are permitted to give. If we give more of oxytocin or Epidosin sometimes there are chances of the uterus getting ruptured, and that is the reason we don’t give more doses…. In the future, if there is excess bleeding, it is the life of two–not one–and so we don’t want to take any decision.” – Bagalkot, Village Doctor*

However, several village doctors also appeared ignorant of potential oxytocin side effects, considering it safe for labor augmentation. Most *dais* interviewed were insistent that they never encountered obstetric complications while attending deliveries. However their descriptions of childbirth cases suggest the presence of unrecognized or untreated complications, such as mention of bleeding that continues for days after delivery.

### Provider Perceptions of Family Members’ Roles in Uterotonic Use

Many providers stated that delivering women and accompanying family members demand uterotonics. Formal providers reported that family members were aware of the effects of uterotonics, perceiving drugs or injections to help in increasing labor pains and speeding up delivery. Physicians suggested that family members see the provision of injections as an indication of active medical care. However, providers reported that family members insist upon injections without being aware of potential adverse effects of excessive uterotonic use. ANMs also suggested that some women present themselves at health facilities on the estimated due date, even if labor has not begun. There is some indication that this leads to induction and augmentation of labor.

*“They would have learned from somebody about the [oxytocin] injection and they would insist us to give.” – Hassan, ANM*

*“Almost all patients ask for it. They [family members] say like that when she is in pain for longer time she cannot bear it, so they say if you have anything to give her or prescribe, we will buy and come. Like that, they [family members] say when she has pain and it increase she will deliver faster.” – Bagalkot, ANM*

*“If the patient comes and if we don’t give injections or drips, they will say you are not doing anything for the patient; the patient is left alone….”– Hassan, Physician*

While providers described feeling pressure from family members, most reported resisting it.

*“No, since they are requesting, we cannot give injections, but we have to convince them why it is unnecessary for injection to be given; and if given also it would create problems for the mother and the baby.” – Bagalkot, Physician*

Some physicians reported using placebos to address family members’ demands for injections to augment labor.

*“If they [family members] are like arrogant and irritating patients who start asking the staff nurse, so if something like that is there, I have instructed them [nurses] not to give, but for their satisfaction give them a placebo or give them some vitamin B complex injection but don’t give Pitocin [trade name for synthetic oxytocin].” – Bagalkot, Physician*

Informal providers also reported pressure from family members to administer uterotonics to augment labor:

*“All of them [family members] asked for it and they don’t specify it by saying prescription and all but generally they say to give more injections so that delivery happens fast. We should give in limit all those injections but what they say is give more quantity, and they [family members] are under the impression that if you give one or more injection, delivery would happen fast, but it’s not like that. You have to give only so many units, and for that to act would take so much time. Normally they force us seeing the condition of the patient, but we have to look after our motive also.” – Hassan, Village Doctor*

### Community Members’ Knowledge and Perceptions Regarding Uterotonics

Some recently delivered mothers did not know why they were given injections during labor. Others reported multiple reasons they believed injections were used during labor: to protect from infection, give strength, and, most frequently, “to increase pain” and facilitate or speed delivery. The linkage between pain and speed of delivery was mentioned by many women, including those who believed that injections during labor might also have other effects.

*“According to me it is given so that there will not be infection and to get more pain.” – Hassan, Mother*

*“I didn’t have much pain so they gave me medicines so that I don’t get tired and I don’t know why they gave other injections.” – Hassan, Mother*

*“When I went there I was getting normal pain at little intervals and then they gave an injection. They gave pain injection to make the pain increase and did the delivery.” – Hassan, Mother*

*“After they gave the injection, I started getting pain immediately and then I delivered…. After they gave it, within half an hour I delivered and I did not feel tired.” – Hassan, Mother*

Recently delivered mothers who perceived the effects of uterotonic injections as increasing pain or facilitating delivery found it acceptable to take injections themselves, and also reported that they would recommend such use to other women. Some women described potential harms in not using injections.

*“As per my knowledge I have told one or two pregnant ladies to take this injection for easy delivery.” – Hassan, Mother*

*“I feel it is good since, for the first child, though I got little pain, it had got late for delivery so then they gave the injection and did the delivery. Like for this lady they made her wait for two days to get the pain but still she did not get any pain and they did not give any injection…. so they did a caesarean…. If they had given injection at that time itself, it would be good…” – Hassan, Mother*

As with recently delivered mothers, most mothers-in-law also identified increasing pain and hastening delivery as the primary purpose of injections during labor. One mother-in-law also specifically discussed the role of injections in PPH treatment.

*“For the baby to come out fast, and after applying that [injection], within a few minutes the pain increased a lot and the baby came out.” – Hassan, Mother-in-law*

*“To increase the pain, they gave five injections. Because of it the pain increased and finally they gave one more injection to stop bleeding.” – Bagalkot, Mother-in-law*

*“Nowadays you don’t have that facility at home, it won’t be clean and hygienic, and no new clothes or no new blade or no new thread will be used, and even dais also are less these days. That’s why hospital is best, and in case delivery happens at home also there might be lot of bleeding and you don’t know what to do at that time, so if you go to hospital if bleeding happens also they know what to do; they give medicines and injections to control it.” – Bagalkot, Mother-in-law*

Many mothers-in-law also suggested that they were physically stronger than their daughters-in-law and thus could bear labor pain and delivery more easily. In comparison, they believed daughters-in-law to be frail and unable to withstand much pain. The use of injections during labor was also discussed in this context, particularly in contrast to traditional preparations used in the past:

*“In those days ladies were strong and so delivery used to happen at home and these days they are not strong; they don’t have the strength to sustain pain so they go to hospital.” – Bagalkot, Mother-in-law*

*“They were using coconut oil and then they will make us bend and put some hot water…. They would give some liquid, kusu and alavi [local fruits/seeds] before the delivery and so by that the child would come out easily. But now they do a caesarean and give injections to start or increase pain.” – Bagalkot, Mother-in-law*

The majority of recently delivered mothers stated that the decision to administer an injection during labor was made by providers, including physicians, nurses, and *dais*. In contrast to provider feedback, women did not report that they or their family members insisted on injections.

*“No, my mother never told anything…. They [nurses at facility] themselves told if she gets the pain we will do normal delivery; otherwise we will do a caesarean, and as I never got the pain, they did a caesarean. They gave an injection in the morning at 10 and I never got the pain.” – Hassan, Mother*

*“The pain was coming on and off and doctor was not there, and duty nurse told that duty doctor will come anytime, in the meantime we admit you, and… once the doctor came they gave me injection to increase the pain and then delivery happened.” – Hassan, Mother*

Mothers-in-law also reported that health providers decided upon use of injections to augment labor.

*“…the doctor said it will be late and it may become late in the evening, so we will give an injection for pain; otherwise, she will get tired. Then they gave two injections to her hips and then they gave a few more injections along with the drips, and they gave three bottles of drips, and after that she got pain and delivered the baby….” – Hassan, Mother-in-law*

*“I could see she was on drips, and since I knew the sisters there, they told me that they had given one injection now so delivery would happen any time, but since she was not able to withstand pain, they called me inside. So I went inside, and when I was going inside I could see them giving one more injection into the drip bottle and they said that it would induce pain fast and delivery would happen, and the reason for doing so is because she was not in a position to bear the pain.” – Hassan, Mother-in-law*

Additionally, mothers-in-law reported that their roles in institutional deliveries were limited to buying medicines requested by providers, particularly when out of stock at public facilities, and getting tea and biscuits for the delivering woman.

## Discussion and Conclusions

### Key Findings

Many of the interviewed providers, both formal and informal, described common use of uterotonics for a variety of purposes–to induce labor, augment labor, prevent PPH, and treat PPH. Based on respondent feedback, it appears that augmenting labor is the most frequent use, although induction also appears to be common. Oxytocin is the most frequently used uterotonic drug. This is consistent with previous studies documenting widespread inappropriate use of uterotonics at home births in India, and with the findings of a recent study of facility and community uterotonic use by the same team in Uttar Pradesh [10], [15]–[16], [34].

Considering the feedback of different respondent cadres together, several notable themes emerge:

#### Pain during childbirth

Both informal providers and community members conflated pain with active, progressing, and/or adequate labor. Many respondents within these cadres described the need to increase pain in order to facilitate delivery, generally achieved through oxytocin injections. Several respondents described women needing caesarean sections because the “pain didn’t come.” This is similar to findings by other researchers in India and South Asia [17], [18]. Given that mothers-in-law frequently described believing that women today could not sustain or handle labor and delivery pain, it is possible that delivering women feel pressure to demonstrate “appropriate” childbirth by taking injections that increase labor pains.

#### Conflicting perspectives on uterotonic demand

While formal providers reported experiencing significant pressure from family members to administer uterotonics, recently delivered women and mothers-in-law described uterotonic use as being decided upon or driven by providers. This difference in perspective may reflect a form of social desirability bias, specifically an unwillingness to acknowledge demanding or providing uterotonics to increase the speed of delivery if this is understood to be an inappropriate or incorrect practice. However, family member reports may also reflect the degree to which uterotonic use, for labor augmentation in particular, has become part of the socio-medical culture, even among clinicians who can correctly identify serious risks from inappropriate use. It is also possible that each set of stakeholders (formal providers and community members) may perceive such use to be the norm for the other group, indicating a need for communication interventions to clarify misconceptions.

#### Inconsistent knowledge and practices among providers

Both health providers and chemists appeared to have incomplete and inconsistent knowledge about uterotonics, including appropriate dosage, required monitoring, and storage requirements. For example, many chemists reported storing various uterotonics without refrigeration, including those for which this is inappropriate. Similarly, even though many clinicians appeared to be aware of the risks of uterotonics, numerous clinician respondents described non-indicated practices such as combined IM/IV administration during the first and second stage of labor. Providers also described changing practices regarding the use of oxytocin in particular, with some providers currently using it only for AMTSL and others using it throughout the first and second stages of labor. Finally, providers described administering high doses of oxytocin for labor augmentation despite expressing an awareness of potential risks. This inconsistency of provider knowledge and practices has implications for training programs.

### Public Health Implications

Numerous efforts are under way in Karnataka to increase institutional deliveries and skilled attendance at birth. Efforts are also ongoing to increase access to uterotonics so that AMTSL can be provided for PPH prevention at both facility and community levels. Additionally, even for community births not attended by SBAs, non-formal providers evidently attempt to imitate what they observe or perceive to be the standards of care for formal providers and at health facilities. As the demand for and utilization of facility-based delivery services and of biomedical interventions at the community level increase, it is essential that quality of care also be improved. In the context of inappropriate use and enabling factors documented through this study, public health programs need to consider:

- Applying approaches (e.g., Client-Oriented, Provider-Efficient Services [COPE] and Partnership-Defined Quality) that bring community members and providers together to discuss their respective perceptions of high-quality care and to address the sources of pressure or motivation to increase labor or speed delivery through uterotonics [32], [33].
- Ensuring that pre-service training uses curricula updated to reflect current guidance regarding uterotonic use, includes opportunities to practice these skills, and includes information about practices to avoid in addition to recommended interventions.
- Providing in-service/refresher training to current providers to ensure alignment with current SBA guidelines, but also to address identified and observed knowledge gaps, misconceptions, and non-indicated practices. This may reduce observed variations in care resulting from the fact that providers with different levels of experience were trained with different guidance related to uterotonic use.
- Developing education and behavior change communication materials for community members and job aids for health providers and chemists to ensure that appropriate uterotonic use is understood and reinforced.

### Strengths and Limitations

A key strength of this study is that by incorporating a wide range of perspectives it was able to generate a comprehensive picture of why and how uterotonics are used during labor and delivery, and how providers and community members perceive this use. For example, the study revealed contradictions between providers’ and community members’ perceptions of uterotonic use, an issue that cannot be uncovered through research targeting only the health system or the community.

The study faced some limitations, including potential recall bias, e.g., if mothers with more traumatic birth experiences or adverse outcomes were more likely to remember receiving injections and to describe these practices. However, it is believed that the use of comprehensive, standardized IDI guides and the restriction of interviews to within six months of delivery mitigated this limitation. Another limitation is that it was not possible to conduct multiple interviews with subjects, which would have enabled probes on specific topics that emerged after interviews with other cadres.

### Conclusion

This study has documented common use of uterotonics, particularly oxytocin, for labor augmentation in two districts of Karnataka. Much described use is inappropriate in the context of global and national guidance for administration of uterotonics. The study has also identified contextual factors that promote inappropriate use, including high valuation of pain during labor; perceived pressure from patients’ families and/or providers to provide or receive uterotonics early in labor and delivery that may lead to administration of uterotonics despite awareness of risks; and lack of consistent knowledge regarding the safe storage, dosing, and administration of oxytocin. These findings have significant implications for public health programs in a context of widespread and potentially increasing availability of uterotonic drugs, including improved communication between medical providers and community members, as well as consistent and expanded pre-service and in-service training.
